# Cerebral Infarction and Evan’s Ratio on MRI Affect the Severity and Prognosis of Tuberculosis Meningitis Patients

**DOI:** 10.3390/diagnostics12051264

**Published:** 2022-05-19

**Authors:** Xin Cao, Qingluan Yang, Xian Zhou, Kun Lv, Zhe Zhou, Feng Sun, Qiaoling Ruan, Jun Zhang, Lingyun Shao, Daoying Geng

**Affiliations:** 1 Department of Radiology, Huashan Hospital, Fudan University, Shanghai 200040, China; 13262566515@163.com (X.C.); lvkun85093@163.com (K.L.); m18764209657@163.com (J.Z.); gdy_2019@163.com (D.G.); 2 Center for Shanghai Intelligent Imaging for Critical Brain Diseases Engineering and Technology Reasearch, Shanghai 200040, China; 3 Department of Infectious Diseases, National Medical Center for Infectious Diseases, Shanghai Key Laboratory of Infectious Diseases and Biosafety Emergency Response, Huashan Hospital, Shanghai Medical College, Fudan University, Shanghai 200040, China; qlyang10@fudan.edu.cn (Q.Y.); zhouxian-13@163.com (X.Z.); 20211220006@fudan.edu.cn (Z.Z.); aaronsf1125@126.com (F.S.); lingyun26@fudan.edu.cn (L.S.); 4 National Clinical Research Center for Aging and Medicine, Huashan Hospital, Fudan University, Shanghai 200040, China

**Keywords:** tuberculosis meningitis, magnetic resonance imaging, cerebral infarction, hydrocephalus, neurological deficits, prognosis

## Abstract

Background: Magnetic resonance imaging (MRI) is widely used in the diagnosis of tuberculous meningitis (TBM) and its complications. We aimed to explore the relationship between MRI features and neurological deficits and TBM patients’ prognosis. Methods: patients diagnosed with TBM were subjected to a neurological evaluation on admission and divided into groups based on the Medical Research Council (MRC) scale. After several years of follow-up, the patients were further divided into groups according to the Modified Rankin Score (MRS). Their MR images were analyzed for meningeal enhancement, tuberculomas, infarction, hydrocephalus, and abscess, including the location and size of the lesion. Any changes in MRI features during the follow-up were recorded. MRI features between groups were compared, and the relationship between dynamic changes in images and Rankin grading was explored. Results: We found significant differences in acute cerebral infarction (ACI) and old cerebral infarctions (OCI) between the MRC groups, and the ORs of ACI and OCI were 21.818 (95% CI: 2.440–195.075) and 6.788 (95% CI: 1.516–30.392), respectively. There were significant differences in ACI, OCI, and Evan’s ratio between the MRS groups (*p* < 0.05), and the ORs of ACI, OCI, and hydrocephalus were 6.375 (95% CI: 1.501–27.080), 5.556 (95% CI: 1.332–23.177), and 9.139 (95% CI: 2.052–40.700), respectively. The changes of Evan’s ratio were related to the MRS grading (r = 0.335, *p* = 0.040). Conclusions: For patients with TBM, the presence of ACI or OCI is associated with neurological deficits, and ACI, OCI, and hydrocephalus can be regarded as poor prognostic predictors. Changes in Evan’s ratio will affect the outcome.

## 1. Introduction

Tuberculous meningitis (TBM) is a potentially fatal infection with risks of complications and severe neurological sequelae despite the availability of effective anti-tuberculous (anti-TB) drugs [1]. TBM accounts for 5–10% of all TB cases and causes 10–30% of deaths, with one-third of survivors experiencing long-lasting sequelae [2,3].

TBM is associated with exudates, tuberculoma, cerebral infarction, abscess, and hydrocephalus, which determine the clinical picture and outcome [4]. Cranial magnetic resonance imaging (MRI) has been widely used for the detection of these signs, which may occur in isolation or in combination. Importantly, the pathogenesis of TBM involves infiltrative, proliferative, and necrotizing processes that impact intracranial vessels [5]. Vasculitis may occur in the vessels traversing the exudates, leading to infarction, which is the main cause of long-term morbidity [6]. Cerebral infarction is a common complication, with estimates ranging between 6% and 47% of all TBM cases [7,8]. MRI is superior to computerized tomography for revealing acute cerebral infarction (ACI), especially diffusion-weighted imaging (DWI), which has a higher sensitivity for the detection and localization of ACI [9]. Hydrocephalus is the most common cause of increased intracranial pressure [3,10], and its severity can be assessed by the measurement of the ventricular dilation width. MRI scanning is valuable in the diagnosis of hydrocephalus because it can measure Evan’s ratio and cerebrospinal fluid (CSF) flow [11]. Tuberculomas are aggregates of immunological and inflammatory cells, present in 16–40% of patients with TBM [2,12], and they are usually considered to be a feature of paradoxical worsening in patients treated for TB [13]. Gadolinium-enhanced MRI is suitable for the visualization of small tuberculomas, with homogeneous nodular contrast enhancement or ring-like enhancement [14].

Although some studies have explored the correlation between the imaging features of TBM and its complications on patient prognosis [1,2,4,15], this study aimed to evaluate the predictors of severe neurological deficits and poor prognosis and to explore the relationship between dynamic changes in MRI characteristics and the prognosis of patients over an eight-year follow-up period. Searching for potential mechanisms may help determine effective treatment options and minimize the disability and mortality associated with TBM.

## 2. Materials and Methods

### 2.1. Study Design and Participants

This retrospective study included patients who were admitted to the department of infectious diseases and diagnosed with TBM in our hospital from January 2010 to January 2019. Patients’ diagnosis of TBM was confirmed by testing positive for Mycobacterium tuberculosis (*M. tb*) in the CSF culture. Patients that were highly probable or probable for TBM were diagnosed according to CSF criteria as well as supporting criteria. The CSF criteria included three parameters: (i) a CSF glucose level of <50%, (ii) a CSF lymphocytes level of >50%, and (iii) a CSF protein level of >1.5 g/L. The supporting criteria contained two parameters: (i) enhanced MRI scan features consistent with TBM and (ii) evidence of extra-central nervous system (CNS) tuberculosis or positive T-SPOT.TB assay results. Highly probable TBM was diagnosed when at least two CSF criterion parameters and two CSF criterion items, or three CSF criterion parameters and one CSF criterion item, were fulfilled. Conversely, probable TBM was diagnosed when two CSF criterion items and one CSF criterion item were fulfilled. A diagnosis of possible TBM was made if patients did not fulfill the above criteria but active TB could not be excluded [16]. Exclusion criteria included (i) patients that were infected by pathogens other than tuberculosis; (ii) patients that also suffered from other CNS diseases, such as a brain tumor or autoimmune encephalitis; (iii) patients that received less than one week of anti-TB treatment in the hospital; (iv) incomplete clinical data and examination; (v) loss of follow-up; and (vi) MR images that had severe motion artifacts that did not meet our research needs.

### 2.2. Evaluation

Each patient’s hospital record was thoroughly reviewed by a trained reviewer for their condition at presentation, clinical symptoms, results of examinations, treatment plan, and outcome at discharge. Patients were assessed by two senior neurologists for the severity of TBM at admission according to the Medical Research Council (MRC) criteria [17]. Stage I: meningitis with no focal neurologic signs; Stage II: meningitis with focal deficits or a Glasgow Coma Scale (GCS) score of 11–14; Stage III: severe alteration of the patients’ sensorium, convulsions, focal neurological deficits, and involuntary movements with a GCS score <11. Patients were assessed for any disability at follow-up in 2019, and their prognosis was assessed using the Modified Rankin Score (MRS). A score of 0 indicated no symptoms, classified as the Rankin 0 group; a score of 1 or 2 indicated favorable prognosis, classified as the Rankin I group; a score >2 indicated severe disability with poor prognosis, classified as the Rankin II group; patients that died were classified as the Rankin III group.

### 2.3. Investigations

CSF was examined for opening pressure, protein expression, white blood cells, glucose, AFB in smear and culture for mycobacteria, and PCR for *M. tb*. MRI was performed in the head-first supine position using 3.0-T MR systems Discovery MR 750, GE Healthcare (Boston, MA, USA) or MAGNETOM Verio, Siemens (Munich, Germany) with 8-channel phased-array head and neck coils. T1W, T2W, fluid-attenuated inversion recovery (FLAIR), DWI, and enhanced T1W images were obtained from the Picture Archiving and Communication System.

### 2.4. Image Analysis

**Figure 1:**
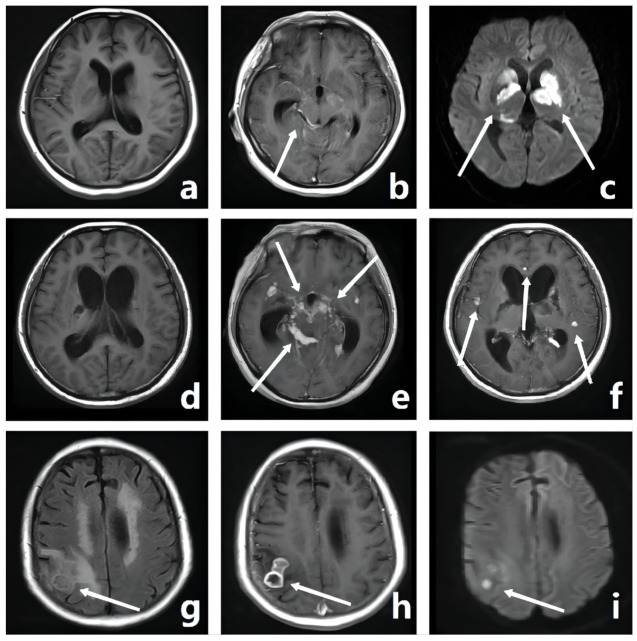
A 26-year-old female. There was exudation in her skull base, and the meninges of the ambient cistern and lateral fissure cistern were enhanced on T1WI ((**b**), white arrow). She had ACI in the bilateral basal ganglia, which showed hyperintensity on DWI ((**c**), white arrows). One month later, the patient was reexamined with MRI. She developed moderate hydrocephalus with an Evan’s ratio of 0.35 (**d**), compared with the initial Evan’s ratio of 0.30 (**a**). There are countless tuberculomas in the meninges ((**e**), white arrows) and brain parenchyma ((**f**), white arrows), showing obvious homogeneous nodular enhancement. Another 70-year-old female. She had several brain abscesses in the right parietal lobe and multiple OCIs in the radiated coronal area ((**g**), white arrow). The abscess wall showed ring-shaped enhancement ((**h**), white arrow), and the abscess cavity showed obvious high signal intensity on DWI ((**i**), white arrow).

The first cranial MRI examination was performed within 24 h of the patient’s initial diagnosis. MRI results were interpreted independently by two senior neuroradiologists who were blinded to the treatment and clinical information. The imaging characteristics of each MRI sequence and the feature information, including location and size to be recorded, are shown in Table 1. ACI volume measurements were based on the high signal intensity of DWI images (Figure 1b) using ITK-SNAP software v.3.6.0 (created by Paul Yushkevich, Ph.D., the University of Pennsylvania, Pennsylvania, USA, and Guido Gerig, Ph.D., the University of Utah, Utah, USA, www.itksnap.org/, 15 August 2021). Diffuse lesions refer to a large number of lesions, which were difficult to accurately count (Figure 1e,f). Hydrocephalus was defined as the presence of ventriculomegaly with an Evan’s ratio (maximal width of frontal horns/maximal width of the inner skull) >0.30 and/or at least one temporal horn >2.0 mm (Figure 1d). The dynamic changes in MRI characteristics were compared between the initial diagnosis and the follow-up re-examination every 3–6 months, and the image features that were measured and compared are shown in Table 1 and Figure 1. Worsening of imaging performance was recorded as “1”, remaining unchanged as “0”, and reduced as “−1”.

### 2.5. Statistical Analysis

Descriptive statistics are reported as medians with interquartile ranges for quantitative variables and as frequencies for categorical variables. The categorical and continuous variables of patients’ MRI features were compared using the *χ*^2^ test and the Mann–Whitney U test, respectively. An odds ratio (OR) and corresponding 95% confidence interval (CI) of MRI features was calculated in order to distinguish the MRC I group from the MRC III group and to distinguish the Rankin 0 group from the Rankin II group. A Spearman’s correlation was used to determine the relationship between dynamic changes in imaging characteristics and the Rankin grading. All statistical analyses were performed with Statistical Package for the Social Sciences v.23.0 (IBM, Armonk, New York, NY, USA). *p* < 0.05 was considered statistically significant.

The study protocol was approved by our hospital’s Institutional Review Board (KY2013-332, 19 April 2014) and a waiver for informed consent was issued given that this study used data collected as part of the participants’ routine care.

## 3. Results

### 3.1. Patients’ Clinical Characteristics

Within the period of this study, 161 patients fulfilling the criteria for TBM were admitted to our hospital. Among them, 32 patients were lost to follow-up, 14 patients did not undergo cranial MRI examination, and five patients had poor quality images due to motion artifacts. Therefore, in total, 110 patients (average age = 43.17 ± 16.90 years; 70 males and 40 females) were included in the analysis. Their initial clinical symptoms and CSF results on admission are shown in Table 2.

### 3.2. Relationship between MRI Features and Neurological Deficits

**Figure 2:**
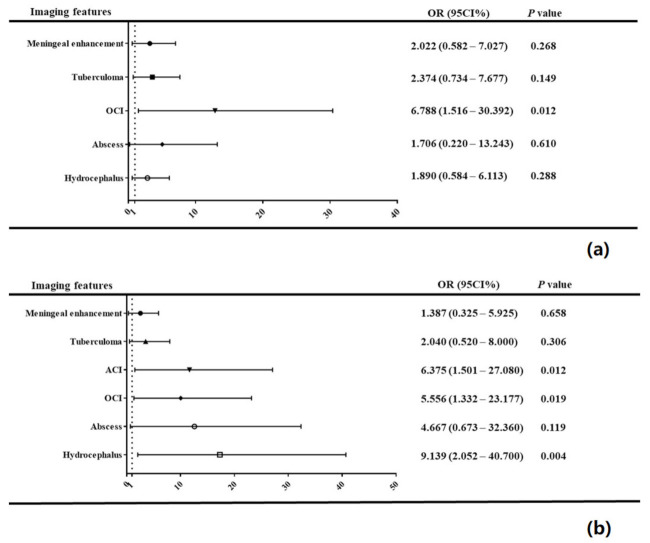
The MRI features as the risk factors for assessing neurological deficits (**a**) and poor prognosis (**b**) in TBM patients.

Out of 109 patients who were evaluated for the MRC TBM grading on admission according to their neurological damage, 31 (28.44%), 59 (54.12%), and 19 (17.43%) cases were classified into the MRC I, II, and III groups, respectively (Table 3). After cranial enhanced MRI, 71 (65.14%) had various ranges of meningeal enhancement, 47 (43.12%) had at least one tuberculoma, 23 (21.10%) had ACI, 18 (16.51%) had old cerebral infarction (OCI), 7 (6.42%) had at least one abscess, and 38 (34.86%) had hydrocephalus. There were significant differences in the meningeal enhancement of the suprasellar cistern and tuberculoma of the corpus callosum between the groups (*p* < 0.05). There were also significant differences in ACI and OCI between the groups (*p* < 0.01), including in frontal lobe ACI (*p* = 0.01), temporal lobe ACI (*p* < 0.01), frontal lobe OCI (*p* < 0.01), parietal lobe OCI (*p* < 0.01), and temporal lobe OCI (*p* < 0.01). The ORs of ACI and OCI were 21.818 (95% CI: 2.440–195.075, *p* = 0.006) and 6.788 (95% CI:1.516–30.392, *p* = 0.012), respectively (Figure 2a). There were also significant differences in Evan’s ratio between the groups (*p* < 0.01). There were no significant differences in the appearance of brain abscesses, diffuse tuberculoma, and ACI volume between the groups.

### 3.3. Relationship between the MRI Features and Prognosis

Out of 108 patients who had been evaluated for MRS grading according to their follow-up CNS symptoms and disabilities, 59 (54.62%), 32 (29.62%), 10 (9.26%), and 7 (6.48%) cases were classified into Rankin 0, I, II, and III groups, respectively (Table 4). After cranial enhanced MRI, 70 patients (64.81%) had various ranges of meningeal enhancement, 46 (42.59%) had at least one tuberculoma, 22 (20.37%) had ACI, 18 (16.67%) had OCI, 7 (6.48%) had at least one abscess, and 36 (33.33%) had hydrocephalus. There were significant differences in ACI, OCI, hydrocephalus, and Evan’s ratio between the groups (*p* < 0.05). The ORs of ACI, OCI, and hydrocephalus were 6.375 (95% CI:1.501–27.080, *p* = 0.012), 5.556 (95% CI:1.332–23.177, *p* = 0.019), and 9.139 (95% CI: 2.052–40.700, *p* = 0.004), respectively (Figure 2b). There were no significant differences in the appearance of meningeal enhancement, tuberculoma, brain abscess, diffuse tuberculoma, and ACI volume between the groups.

### 3.4. Relationship between the Dynamic Changes in MRI Features and Prognosis

A total of 68 (61.81%) patients had more than one MRI examination. They had a follow-up visit every 3–6 months for several years after discharge, and 5 patients died. By comparing the imaging features of multiple examinations, changes in Evan’s ratio were found to be related to the MRS grading (r = 0.335, *p* = 0.040). When the Evan’s ratio increased, the patient’s MRS grading increased, and their prognosis became worse. However, other imaging features, including changes in the size and number of tuberculoma or abscess, and the scope of meningeal enhancement were not associated with the patient’s MRS grading.

## 4. Discussion

This study investigated the relationship between MRI features of TBM and the severity of neurological deficits and patients’ prognosis. We found that the presence of cerebral infarction was a predictor of neurological impairment and that the presence of cerebral infarction or hydrocephalus were predictors of a poor outcome in terms of functional disability. Furthermore, during an average follow-up of four years, the Evan’s ratio was also a factor affecting the prognosis. A patient’s future outcome can be judged from these imaging features, allowing for an adjustment to their treatment plan to maximize the recovery of their neurological function.

Cerebral infarcts were present in 30.00% of patients, out of which 69.69% were acute and 54.54% were old, with some presenting both. The predominant basal exudate which surrounds the circle of Willis causes arteritis, which may result in vasculitis, thrombosis, and vasospasm, with the middle cerebral arteries and their perforating branches to the basal ganglia bearing the brunt of this [18]. The association between poor prognosis and cerebral infarcts in TBM patients has been previously documented [7,19,20,21], and this relationship was further verified in this study. One meta-analysis, which included 21 studies describing factors associated with death from TBM, found that cerebral infarction (OR = 2.35, 95% CI: 1.63–3.38) was one of the most significant prognostic indicators [21]. We further distinguished the different stages of cerebral infarction and found that the presence of ACI (OR = 6.38, 5% CI: 1.50–27.08) or OCI (OR = 5.56, CI: 1.332–23.177) was a predictor of poor prognosis. It is important to note that OCI should not be ignored, because it indicates that the patient has a history of stroke. Patients with a poor cerebrovascular condition are prone to repeated strokes, and once infected with *M. tb* and suffering from TBM, there is a greater possibility of disability, even death. Certain areas of the brain also have a predilection for the development of infarctions. By summarizing the infarction location, it was found to be consistent with the blood supply area of the middle cerebral arteries, and the occipital lobe and cerebellum were almost unaffected, which was similar to previous reports [1,22]. However, we found that only 22.72% of cases had infarctions in the basal ganglia and thalamus, which may suggest another possible non-inflammatory ischemic mechanism in these cases [23]. We also measured the volume of TBM patient’s ACI and found no relationship with the neurological deficits and their prognosis, indicating that the location of the infarction was more important than the volume and that the effects of infarction in different brain regions is not equal. Nevertheless, death and disability related to TBM could be substantially reduced by preventing cerebral infarctions. As a large, randomized, placebo-controlled trial showed, regardless of the severity of TBM, early dexamethasone therapy is effective in reducing mortality [24]. Therefore, combining aspirin with dexamethasone treatment may improve outcomes from TBM [25], but its role as a preventive therapy is still being investigated [10,13,20,26]. More clinical trials and research need to be carried out to study the exact etiology and pathology of infarction in TBM patients.

Hydrocephalus was present in 38 (34.54%) patients, and the average Evan’s ratio was 0.27 in this study. Communicating hydrocephalus is caused by exudates in the basal cisterns and resulting in disruption of CSF flow. On the other hand, noncommunicating hydrocephalus, caused by obstruction at the level of the cerebral aqueduct and/or the outlet foramina of the fourth ventricle, is less common in this population. Previous studies have found that hydrocephalus is a determinant of poor outcome and mortality in patients with TBM [27,28], which was verified in this study. However, the neurological deficits in TBM patients with hydrocephalus were not significantly different from those without hydrocephalus at the time of admission. Therefore, attention should be paid to the treatment of hydrocephalus, even if the patient does not initially show severe CNS symptoms. Hydrocephalus can develop early in the course of the disease or paradoxically during antituberculosis treatment. During a mean follow-up of four years, the Evan’s ratio increased in four cases and decreased in 16 cases. The prognosis of the patients worsened with the aggravation of hydrocephalus. It is possible that if the increased intracranial pressure goes untreated, it can result in an alteration in consciousness and a stretching of compromised vessels, which could cause an infarction. Moreover, the optic nerve can become compressed by the enlarged dilated third ventricle, resulting in vision loss and optic atrophy [27].

Tuberculoma was present in 43.63% of patients, including 27 with brain parenchymal tuberculomas, 39 with meningeal tuberculomas, and some with both. The presence of tuberculomas can cause infarction and inflammatory adhesive exudates, which may then result in obstructive hydrocephalus and multiple cranial nerve palsies [2]. Although most studies suggest that tuberculomas are not associated with long-term relapse or poor clinical outcomes [10,15], data regarding prognostic value have been limited by conflicting evidence [29]. In this study, the presence of tuberculoma had no adverse effect on the outcome, neither the location nor the quantity of tuberculoma was a factor determining CNS damage, and changes in the number or size of tuberculoma will likely not affect the patient’s prognosis. Increasing evidence suggests that TBM patients with tuberculoma can obtain a good prognosis via aggressive and prompt treatment.

The meningeal enhancement of the suprasellar cistern and the tuberculoma in the corpus callosum were related to the patient’s MRC grading, which might be due to the vision impairment secondary to tuberculosis of the optic chiasma region and the damage to the corpus callosum, respectively. However, these findings have to be further confirmed in large, multi-center trials. Brain abscess is a rare complication of TBM [30]; a total of seven (6.36%) cases were found in this study, which was of no significance to clinical grading.

The present study had limitations. Many patients underwent only one brain MRI scan on admission, resulting in a reduction in the sample size that could reflect the changes in MRI characteristics at different periods. Furthermore, due to the limited number of patients with ACI, the 95% CI of OR for the MRC III group was extremely wide. However, this does not change the conclusion that ACI may still be a risk factor for neurological deficits in TBM patients. Further studies of the combination of basic and advanced MRI sequences are essential for improved the diagnosis ability and accuracy [31].

## 5. Conclusions

For TBM patients, cerebral infarction is associated with neurological deficits, and cerebral infarction and hydrocephalus can be regarded as poor prognostic predictors. Regular follow-up and reexamination of cranial MRI, especially paying more attention to the dynamic change of the Evan’s ratio, are conducive to timely adjustment of the treatment plan, improving their prognosis, and minimizing mortality.

## Figures and Tables

**Table 1:** The characteristics of TBM and its complications on each MRI sequence image and the feature information to be recorded.

	T1WI	T2WI	FLAIR	DWI	Enhanced T1WI	Information to Be Recorded
**Meningeal enhancement**	/	/	/	/	Linear enhancement along the meninges	Location: suprasellar cistern, ambient cistern, lateral fissure cistern, interhemispheric fissure cistern, tentorium cerebelli, spinal cord membrane, and other meninges
**Tuberculoma**	Nodules with equal or slightly low signal intensity	The liquefaction part of the nodule shows high signal intensity, and the non-liquefied caseous necrosis part shows low signal intensity	The liquefaction part of the nodule center shows low signal intensity, the non-liquefied caseous necrosis part shows equal signal intensity, and the tumor wall shows high signal intensity	Low signal intensity	Uniform or circular enhancement	Location: frontal lobe, parietal lobe, temporal lobe, occipital lobe, corpus callosum, basal ganglia, cerebellum, brain stem, and meninges Is the lesion diffuse?
**ACI**	Low signal intensity	High signal intensity	High signal intensity	High signal intensity	Enhanced lesion area	Location: frontal lobe, parietal lobe, temporal lobe, and occipital lobe Volume of infarcted brain tissue
**OCI**	Low signal intensity	High signal intensity	Low signal intensity	Low signal intensity	No enhancement in lesion area	Location: frontal lobe, parietal lobe, temporal lobe, and occipital lobe
**Abscess**	The abscess cavity shows low signal intensity, and the abscess wall shows equal signal intensity	The abscess cavity shows high signal intensity, and the abscess wall shows slightly high signal intensity	The abscess cavity shows slightly low signal intensity, and the abscess wall shows slightly high signal intensity	The abscess cavity shows high signal intensity	The abscess wall shows obvious circular enhancement	Does the patient have a brain abscess?
**Hydrocephalus**	Enlargement of the ventricles without widened sulci					Does the patient have hydrocephalus?
**Lesion size**	Including the scope of meningeal enhancement and the size of tuberculoma and abscess					Increase in size was recorded as “1”, unchanged as “0”, and decrease as “−1”
**Quantity of lesion(s)**	Including the number of tuberculoma and abscess					Increase in quantity was recorded as “1”, unchanged as “0”, and decrease as “−1”
**Severity of hydrocephalus**	Reflected by Evan’s ratio					Increase in the Evan’s ratio was recorded as “1”, unchanged as “0”, and decrease as “−1”

**Table 2:** Clinical characteristics of the study’s cohort.

TBM Diagnosis (n, %)	N = 110
**Confirmed**	33 (30.00%)
**Highly probable**	55 (50.00%)
**Probable**	22 (20.00%)
**Age (yrs)**	43.17 ± 16.90
**Gender (n, %)**	
**Male**	70 (63.64%)
**Female**	40 (36.36%)
**Clinical symptoms** **(n, %)**	
**Headache**	18 (16.36%)
**Fever**	39 (35.45%)
**Focal neurological signs**	35 (31.82%)
**Altered consciousness**	46 (41.82%)
**CSF results on admission**	
**Glucose, mmol/L**	0.28 ± 0.10
**Protein, mg/L**	2061.00 (1260.00, 3519.00)
**WBC,** **×** **10^6^/L**	120.00 (40.00, 316.00)
**MRC grading (n, %) N = 109**	
**I**	31 (28.44%)
**II**	59 (54.13%)
**III**	19 (17.43%)
**Outcome/MRS (n, %) N = 108**	
**0**	59 (54.63%)
**1**	25 (23.15%)
**2**	7 (6.48%)
**3**	6 (5.56%)
**4**	4 (3.70%)
**6**	7 (6.48%)

CSF, cerebrospinal fluid; WBC, white blood cell; MRC, Medical Research Council; MRS, Modified Rankin Scale.

**Table 3:** MRI features of TBM patients with different degrees of neurological deficit.

	MRC I (n = 31)	MRC II (n = 59)	MRC III (n = 19)	*p* Value
**Meningeal enhancement**	18 (58.06)	39 (66.10)	14 (73.68)	0.52
**suprasellar cistern**	8 (25.81)	16 (27.12)	11 (57.89)	0.03
**ambient cistern**	14 (45.16)	25 (42.37)	10 (52.63)	0.74
**lateral fissure cistern**	7 (22.58)	19 (32.20)	10 (52.63)	0.09
**interhemispheric fissure cistern**	3 (9.68)	15 (25.42)	5 (26.32)	0.81
**tentorium cerebelli**	5 (16.13)	17 (28.81)	7 (36.84)	0.23
**spinal cord membrane**	10 (32.26)	15 (25.42)	10 (52.63)	0.09
**other meninges**	5 (16.13)	20 (33.90)	9 (47.37)	0.06
**Tuberculoma**	13 (41.94)	22 (37.29)	12 (63.16)	0.14
**brain parenchymal tuberculoma**	11 (35.48)	17 (28.81)	11 (57.89)	0.07
**frontal lobe**	7 (22.58)	12 (20.34)	8 (42.11)	0.15
**parietal lobe**	6 (19.35)	9 (15.25)	7 (36.84)	0.12
**temporal lobe**	5 (16.13)	12 (20.34)	8 (42.11)	0.08
**occipital lobe**	2 (6.45)	10 (16.95)	5 (26.32)	0.16
**corpus callosum**	1 (3.23)	3 (5.08)	4 (21.05)	0.04
**basal ganglia**	3 (9.68)	7 (11.86)	6 (31.58)	0.07
**cerebellum**	8 (25.81)	12 (20.34)	6 (31.58)	0.58
**brain stem**	5 (16.13)	8 (13.56)	6 (31.58)	0.19
**meningeal tuberculoma**	8 (25.81)	14 (23.73)	4 (21.05)	0.93
**diffuse tuberculoma**	3 (9.68)	9 (15.25)	5 (26.32)	0.29
**maximum diameter**	6.30	5.35	4.80	0.38
**ACI**	1 (3.23)	14 (23.73)	8 (42.11)	0.00
**frontal lobe**	1 (3.23)	5 (8.47)	6 (31.58)	0.01
**parietal lobe**	1 (3.23)	4 (6.78)	4 (21.05)	0.07
**temporal lobe**	0 (0.00)	2 (3.39)	5 (26.32)	0.00
**volume (cm^3^)**	0.65	0.54	1.35	0.140
**OCI**	3 (9.68)	7 (11.86)	8 (42.11)	0.00
**frontal lobe**	2 (6.45)	3 (5.08)	6 (31.58)	0.00
**parietal lobe**	1 (3.23)	3 (5.08)	6 (31.58)	0.00
**temporal lobe**	1 (3.23)	1 (1.69)	5 (26.32)	0.00
**Abscess**	2 (6.45)	3 (5.08)	2 (10.53)	0.70
**Hydrocephalus**	10 (32.26)	19 (32.20)	9 (47.37)	0.45
**Evan’s ratio**	0.26	0.27	0.29	0.00

MRC, Medical Research Council; ACI, acute cerebral infarction; OCI, old cerebral infarction.

**Table 4:** MRI features of TBM patients with different prognosis.

	Rankin 0 (n = 59)	Rankin I (n = 32)	Rankin II (n = 10)	Rankin III (n = 7)	*p* Value
**Meningeal enhancement**	37 (62.71)	21 (65.63)	7 (70.00)	5 (71.43)	0.95
**Tuberculoma**	25 (42.37)	13 (40.63)	6 (60.00)	2 (28.57)	0.60
**meningeal tuberculoma**	17 (28.81)	5 (15.63)	2 (20.00)	1 (14.29)	0.48
**brain parenchymal tuberculoma**	19 (32.20)	11 (34.38)	5 (50.00)	2 (28.57)	0.73
**diffuse tuberculoma**	7 (11.86)	5 (15.63)	3 (30.00)	0 (0.00)	0.31
**maximum diameter**	6.10	4.50	5.50	7.65	0.41
**ACI**	8 (13.56)	6 (18.75)	5 (50.00)	3 (42.86)	0.03
**frontal lobe**	2 (3.39)	3 (9.38)	3 (30.00)	3 (42.86)	0.00
**parietal lobe**	1 (1.69)	3 (9.38)	2 (20.00)	3 (42.86)	0.00
**temporal lobe**	1 (1.69)	1 (3.13)	4 (40.00)	1 (14.29)	0.00
**volume (cm^3^)**	0.62	0.59	0.50	0.69	0.865
**OCI**	9 (15.25)	3 (9.38)	5 (50.00)	1 (14.29)	0.03
**Abscess**	3 (5.08)	2 (6.25)	2 (20.00)	0 (0.00)	0.30
**Hydrocephalus**	12 (20.34)	11 (34.38)	7 (70.00)	6 (85.71)	0.00
**Evan’s ratio**	0.26	0.27	0.29	0.29	0.00

ACI, acute cerebral infarction; OCI, old cerebral infarction.

## Data Availability

The data presented in this study are available on request from the corresponding author. The data are not publicly available due to protecting patient privacy.
